# Clinical and biomarker analyses of hepatic arterial infusion chemotherapy plus lenvatinib and PD-1 inhibitor for patients with advanced intrahepatic cholangiocarcinoma

**DOI:** 10.3389/fimmu.2024.1260191

**Published:** 2024-02-07

**Authors:** YeXing Huang, ZeFeng Du, Anna Kan, MinKe He, HuiFang Li, ZhiCheng Lai, DongSheng Wen, LiChang Huang, QiJiong Li, Li Xu, Ming Shi

**Affiliations:** Department of Hepatobiliary Oncology, Sun Yat-sen University Cancer Center, State Key Laboratory of Oncology in South China, Guangdong Provincial Clinical Research Center for Cancer, Guangzhou, China

**Keywords:** intrahepatic cholangiocarcinoma, hepatic arterial infusion chemotherapy, lenvatinib, PD-1 inhibitor, whole exome sequencing, predictive biomarkers, tumor mutation burden, tumor-infiltrating lymphocytes

## Abstract

**Background:**

Intrahepatic cholangiocarcinoma (iCCA) is a highly aggressive cancer with a dismal prognosis and few effective therapeutic approaches. This study aimed to investigate the efficacy, safety, and predictive biomarkers of hepatic arterial infusion chemotherapy (FOLFOX-HAIC) in combination with lenvatinib and PD-1 inhibitor for patients with advanced iCCA.

**Methods:**

Locally advanced or metastatic iCCA patients receiving the triple combination therapy of lenvatinib, PD-1 inhibitor, and FOLFOX-HAIC were included in this retrospective study. Primary endpoint was the progression-free survival, evaluated using the RECIST criterion. The secondary endpoints included overall survival, objective response rate, and safety. Whole exome and RNA sequencing of tumor biopsy tissues were performed for biomarker exploration.

**Results:**

Between May, 2019 and December 2022, a total of 46 patients were included in this study. The primary endpoint showed a median progression-free survival of 9.40 months (95% CI: 5.28-13.52), with a 6-month progression-free survival rate of 76.1%. The median overall survival was 16.77 months (95% CI, 14.20-19.33), with an objective response rate of 47.8% and disease control rate of 91.3% per RECIST. In addition, 4.3% and 8.7% of patients achieved complete response of all lesions and intrahepatic target lesions per mRECIST, respectively. The most common treatment-related adverse events were neutropenia, thrombocytopenia, elevated aspartate aminotransferase and alanine aminotransferase level. Furthermore, integrated analysis of genetic, transcriptomic, and immunohistochemistry data revealed that pre-existing immunity (high expression level of immune-related signatures and intra-tumoral CD8^+^ T cell density) in baseline tumor tissues was associated with superior clinical benefits. However, the evaluation of tumor mutation burden did not show potential predictive value in this triple combination.

**Conclusion:**

FOLFOX-HAIC in combination with lenvatinib and PD-1 inhibitor demonstrated a promising antitumor activity with manageable safety profiles in patients with advanced iCCA. Moreover, our study also revealed new perspectives on potential biomarkers for clinical efficacy.

## Introduction

Intrahepatic cholangiocarcinoma (iCCA) is the second most common primary hepatic malignancy after hepatocellular carcinoma (HCC), accounting for approximately 10-20% of all newly diagnosed hepatobiliary neoplasms (1–3). Despite the improvements in surveillance of iCCA, the majority of patients are diagnosed at advanced disease stage, and the prognosis of untreated patients is extremely poor, with a median overall survival of 3-6 months (4, 5). Gemcitabine plus cisplatin (GemCis) is currently recommended as a standard treatment regimen for locally advanced and/or metastatic iCCA, which yields a median overall survival of 11.7 months (6, 7). However, treatment options and efficacy remain limited by chemo-refractory, while studies on potential systemic treatments are less well described and of limited effectiveness (8, 9).

Cancer immunotherapy with immune checkpoint inhibitors (ICIs), most notably anti-PD-1/PD-L1 antibodies, have gained success in a spectrum of advanced malignancies, including HCC (10, 11). Recent evidence suggested that chemotherapy in combination with ICIs, such as durvalumab plus GemCis, or camrelizumab combined with gemcitabine plus oxaliplatin (GEMOX), significantly improved the clinical outcomes in advanced biliary tract cancers (BTCs) (12, 13). Lenvatinib is an oral tyrosine kinase inhibitor (TKI) that targets vascular endothelial growth factor receptor (VEGFR) and fibroblast growth factor receptor (FGFR) (14), which reverts VEGF-driven immunosuppression and thereby augments the antitumor activity of PD-1 inhibitor (15). Several preliminary studies further showed that lenvatinib and PD-1 inhibitors displayed synergistic antitumor activity in patients with advanced iCCA (16, 17).

Besides the systemic treatments, locoregional therapies such as radioembolization, transarterial chemoembolization (TACE), and hepatic arterial infusion chemotherapy (HAIC), have also been performed in the treatment for patients with unresectable iCCA. Previous studies showed that hepatic arterial infusion chemotherapy with oxaliplatin, 5-fluorouracil, and leucovorin (FOLFOX-HAIC) yielded superior clinical outcomes to TACE for advanced iCCA (18). By achieving more extensive tumor necrosis and releasing tumor antigens, FOLFOX-HAIC could induce anti-tumor immune response and exert a synergistic anticancer effect with PD-1 inhibitors (19, 20).

Considering the different anti-malignancy mechanisms and the synergistic effects of TKIs, ICIs, and FOLFOX-HAIC, we hypothesize that locoregional treatment combined with systemic therapy might be suitable as a novel treatment option for locally advanced and/or metastatic iCCA. Although efforts have been made to reveal specific genetic and immunologic characteristics to determine the prognostic values for immune-combined therapy, favorable biomarkers are still lacking. Herein, we conducted this retrospective study to evaluate the efficacy, safety, and potential predictive biomarkers of FOLFOX-HAIC in combination with lenvatinib and PD-1 inhibitor for advanced iCCA.

## Materials and methods

### Study design and patients

This was a retrospective study assessing the efficacy and safety of FOLFOX-HAIC in combination with lenvatinib and PD-1 inhibitor as a first-line treatment for advanced iCCA. The trial was conducted in accordance with the Declaration of Helsinki and was approved by the Institutional Review Board of Sun Yet-sen University Cancer Center. Written informed consent was provided by all patients before treatment.

Consecutive iCCA patients receiving FOLFOX-HAIC, lenvatinib and PD1 inhibitor as first-line treatment at Sun Yet-sen University Cancer Center between May, 2019 and December, 2022 were identified. Eligible patients were 18 years or older and diagnosed with unresectable iCCA according to the American Association for the Study of Liver Diseases practice guidelines (21, 22). Other key eligibility criteria for inclusion included the following: no previous systemic treatments for iCCA; Eastern Cooperative Oncology Group performance status (ECOG-PS) of 0-1; Child-Pugh class A liver function; at least one measurable tumor lesions according to the Response Evaluation Criteria in Solid Tumors (RECIST) v1.1 (23); and adequate organ function: neutrophil count >1.2×10^9^ per L, platelet cell count ≥ 75×10^9^ per L, total bilirubin ≤ 30 mmol/L, albumin ≥ 30 g/L, ALT and AST ≤ 5 times upper limit of normal range, and creatinine clearance rate of ≤ 1.5 times the upper limit of the normal range. Exclusion criteria included the following: combined with other malignant tumors; deficient blood supply of tumor indicated from CT or MRI arterial phase; incomplete medical information; and loss to follow-up.

### Treatment protocol

Eligible patients received lenvatinib orally once daily (8mg or 12mg for body weight < 60kg or ≥ 60kg, respectively), as well as a PD-1 inhibitor intravenously every 3 weeks. Every patient was informed of the clinical efficacy, adverse events and cost of each PD-1 inhibitor. The final decision was principally made by patients based on their financial conditions and safety considerations. Five different PD-1 inhibitors were utilized based on patient preference (sintilimab 200 mg, toripalimab 240 mg, camrelizumab 200 mg, pembrolizumab 200 mg, tislelizumab 200 mg). FOLFOX-HAIC was performed every 3 weeks as described in our previous study: a catheter/microcatheter was placed in the main feeding hepatic artery, and then the following regimen was administered via the hepatic artery (24): oxaliplatin 85 mg/m^2^, leucovorin 400 mg/m^2^, 5-fluorouracil 400mg/m^2^ on Day 1, and 5-fluorouracil 2400 mg/m^2^ over 24 hours on Days 1-2. Once FOLFOX-HAIC intolerance occurred, or at end of 6 cycles, the other treatments (lenvatinib and/or PD-1 inhibitors) continued as maintenance therapy until disease progression, deaths or intolerable toxicities.

### Data collection and assessment

Clinical information, laboratory and radiological data were retrospectively collected via the medical records. Tumor response assessment was conducted based on computed tomography (CT) and magnetic resonance imaging (MRI). All radiological data were independently assessed by two radiologists according to RECIST criteria. If there was a controversy, the final judgment was made by another more experienced radiologist.

The primary endpoint was progression-free survival (PFS), which was defined as the interval from treatment initiation to disease progression according to RECIST criteria or death, whichever occurred first. The secondary endpoints included overall survival (OS, defined as the interval from treatment initiation to death from any cause), objective response rate [ORR, defined as the proportion of patients of complete response (CR) or partial response (PR) based on RECIST criteria], disease control rate [DCR, defined as the proportion of patients with CR/PR plus stable disease (SD)], and safety. Treatment-related adverse events assessments were conducted according to the National Cancer Institute Common Terminology Criteria for Adverse Events v4.03.

### Biomarker exploration

To identify potential biomarkers, biopsy samples of tumor tissues were collected and frozen before treatments. Whole-genome transcriptome profiling of biopsy tissues was performed by whole exome sequencing (WES) and RNA sequencing (RNAseq). Briefly, DNA and RNA in tumor biopsy tissues were extracted. Then library preparation, whole exome and RNA sequencing were performed following the standard protocol according to the manufacturer’s instructions. Somatic mutations, including point mutations, small insertions, and deletions, were identified. Tumor mutation burden (TMB) measurement was considered single nucleotide variants, insertions and deletions in the coding region. TMB high was defined as the top 50% value. Pre-treatment tumor biopsies were stored in formalin-fixed paraffin-embedded blocks. Baseline tumor-infiltrating CD8^+^ T cells were evaluated by immunohistochemistry (IHC) staining.

### Statistical analysis

Baseline characteristics and efficacy data were calculated with appropriate methods including the Student’s t-test, the Mann-Whitney U-test, the Chi-square test, or Fisher’s exact test. OS and PFS with associated 95% confidence intervals (CIs) were analyzed using the Kaplan-Meier method, and differences in the survival curves were analyzed using the log-rank test. A *p*-value<0.05 was considered statistically significant. SPSS and GraphPad Prism were used for statistical analysis and bioinformatic analysis of the RNA-seq and WES data was performed using R 4.2.2 software.

## Results

### Baseline characteristics

**Figure 1:**
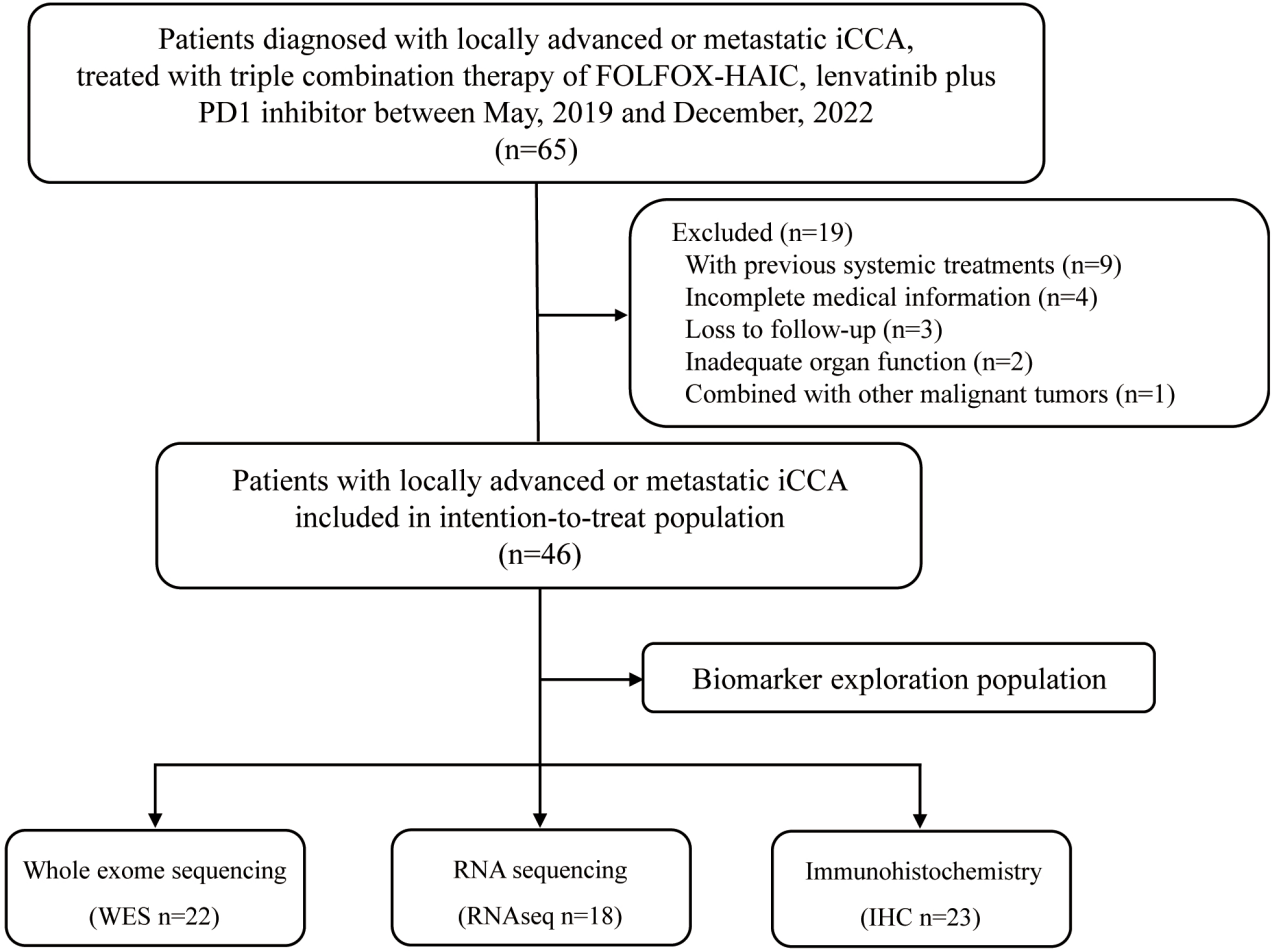
Trial profile. Flow diagram of participants in the study.

Between May, 2019 and December 2022, 65 consecutive patients who received triple combination therapy of FOLFOX-HAIC, lenvatinib plus PD-1 inhibitor were identified, and 46 iCCA patients met the criteria for inclusion in this study (**Figure 1**). The baseline demographic and clinical characteristics of enrolled patients are summarized in **Table 1**. Of the 46 patients included in this study, 23 (50.0%) were male, with a median age of 54.0 years (interquartile range, IQR: 46.3-59.0). In addition, all patients were diagnosed with locally advanced or metastatic diseases, including 80.4% with extrahepatic spread, and 45.7% of patients with macrovascular invasion. Moreover, 41 (89.1%) patients had multiple hepatic lesions and the median size of the maximum lesion was 9.4 cm (IQR: 7.5-12.8). Seventy-six percent of patients had elevated levels of carbohydrate antigen 19-9 (CA19-9, >35 U/mL).

**Table 1:** Baseline demographic and clinical characteristics.

	This Cohort (n=46)
Age, year median (IQR)	54.0 (46.3-59.0)
Sex	
male	23 (50.0%)
female	23 (50.0%)
Etiology	
HBV	20 (43.5%)
Other	26 (56.5%)
ECOG	
0	34 (73.9%)
1	12 (26.1%)
Child Pugh score	
A5	32 (69.6%)
A6	14 (30.4%)
Cirrhosis	
Yes	11 (23.9%)
No	35 (76.1%)
TNM stage at baseline	
IIIA	9 (19.6%)
IIIB	22 (47.8%)
IV	15 (32.6%)
Tumor diameter, cm	
Mean ± SD	10.1 ± 3.3
Median (IQR)	9.4 (7.5-12.8)
Tumor number	
1-3	5 (10.9%)
>3	41 (89.1%)
Vascular Invasion	
Absent	25 (54.3%)
Present	21 (45.7%)
Extrahepatic Metastasis	
Absent	9 (19.6%)
Present	37 (80.4%)
Lymph nodes only	22 (47.8%)
Organ only	1 (2.2%)
Organ plus lymph nodes	14 (30.4%)
CA19-9 level, Median (IQR), U/mL	134.7 (36.7-748.5)
≤ 35	11 (23.9%)
> 35	35 (76.1%)
CEA level, Median (IQR), ng/mL	3.8 (2.3-13.7)
≤ 5	28 (60.9%)
> 5	18 (39.1%)

### Treatment

Treatment administration is listed in **Table 2**. Forty-six enrolled patients were treated with a total of 149 cycles of FOLFOX-HAIC [median (IQR): 3.0 (2.0-4.0)]. In addition, the median treatment cycles of PD-1 inhibitors and duration of lenvatinib were 8.0 (IQR: 5.3-12.0) and 10.4 (IQR: 6.7-15.0) months, respectively. At the date of data cutoff, 7 (15.2%) patients continued maintenance therapy with lenvatinib or PD-1 inhibitors, and were still free from disease progression. After termination of the study treatments, 32 patients received the second-line treatments, including systemic chemotherapy, curative surgical resection, TACE, radiotherapy, and other TKIs plus PD-1 inhibitors. Notably, radical resection was done in three patients with locally advanced iCCA after shrinkage of primary tumor and downgrading, all three of whom survived.

**Table 2:** Treatment Administration.

	This Cohort (n=46)
Study treatment	
Time of lenvatinib (months)	
Mean ± SD	11.6 ± 7.9
Median (IQR)	10.4 (6.7-15.0)
Cycles of FOLFOX-HAIC, median (IQR)	3.0 (2.0-4.0)
Cycles of PD1 inhibitor, median (IQR)	8.0 (5.3-12.0)
PD1 inhibitors	
Sintilimab	18
Toripalimab	11
Camrelizumab	10
Tislelizumab	5
Pembrolizumab	2
Post-study treatment	
Resection	3
Chemotherapy	18
TACE	8
Radiotherapy	5
Other TKIs	14
Other PD1 inhibitors	13

### Efficacy

**Figure 2:**
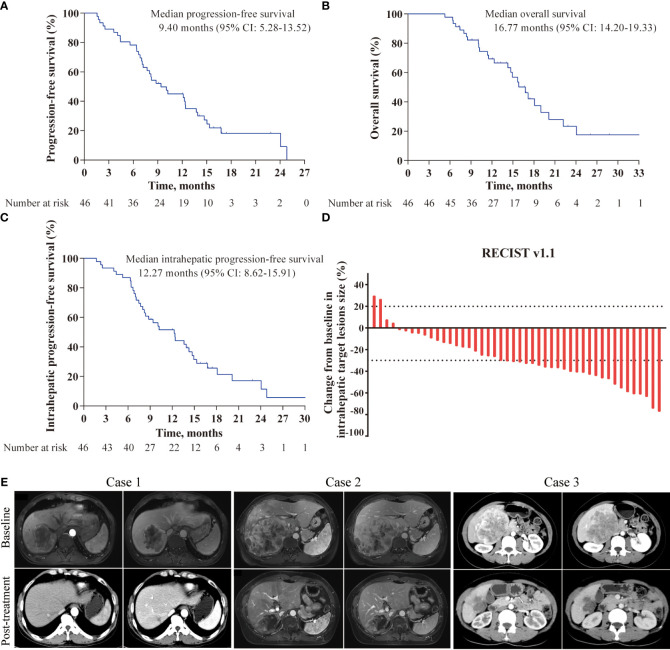
Kaplan-Meier curves and characteristics of tumor response in the study. **(A)** Kaplan-Meier survival curves of progression-free survival; **(B)** Kaplan-Meier survival curves of overall survival; **(C)** Kaplan-Meier survival curves of intrahepatic progression-free survival; **(D)** Best percentage changes from baseline in size of the intrahepatic target lesions; **(E)** Representative images of three patients responded to the triple combination therapy.

At the data cutoff for the analysis (October 1, 2023), all 46 patients completed follow-up, and a total of 37 patients had experienced disease progression or died. In regards to the primary endpoint, the median PFS was 9.4 months (95% CI: 5.28-13.52, **Figure 2A**), with a 6-month PFS rate of 76.1%. The median OS was 16.77 months (95% CI, 14.20-19.33, **Figure 2B**), with a 12-month OS rate of 69.3%. The tumor responses are showed in **Table 3**. Among the 46 examined patients who underwent tumor response evaluation with radiologic imaging, 22 (47.8%) of patients achieved partial response according to RECIST v1.1, 20 (43.5%) had stable disease, 4 (8.7%) experienced progressive disease, with an ORR of 47.8% and DCR of 91.3%. In addition, based on the mRECIST criteria, two patients achieved complete responses, the ORR and DCR were 56.5% and 91.3%, respectively.

**Table 3:** Tumor Response (n=46).

	Overall lesions		Intrahepatic lesions	
	RECIST v1.1	mRECIST	RECIST v1.1	mRECIST
Complete response	0 (0)	2 (4.3%)	0 (0)	4 (8.7%)
Partial response	22 (47.8%)	24 (52.2%)	24 (52.2%)	25 (52.3%)
Stable disease	20 (43.5%)	16 (34.8%)	20 (43.5%)	15 (32.6%)
Progressive disease	4 (8.7%)	4 (8.7%)	2 (4.3%)	2 (4.3%)
Objective response rate	22 (47.8%)	26 (56.5%)	24 (52.2%)	29 (63.0%)
Disease control rate	42 (91.3%)	42 (91.3%)	44 (95.7%)	44 (95.7%)

Given that FOLFOX-HAIC is a locoregional therapy and has better control for liver lesions, tumor responses of intrahepatic target lesions were sequentially tested. The best response rate of intrahepatic lesions was 52.2% per RECIST and 63.0% per mRECIST, with a DCR of 95.7%, including four patients experienced complete response based on mRECIST criteria. The median intrahepatic PFS was 12.27 months (95%CI, 8.62-15.91, **Figure 2C**). A waterfall plot was constructed to show the changes from baseline in the intrahepatic target lesions (**Figure 2D**). In addition, contrast-enhanced CT or MRI scans of three representative patients who received this triple combination therapy are shown in **Figure 2E**.

### Safety

Treatment-related deaths did not occur in this study, and treatment-related adverse events (TRAEs), which occurred in at least 5.0% of patients, are shown in **Table 4**. The common TRAEs were neutropenia, thrombocytopenia, abdominal pain, nausea, fatigue, vomiting, and elevated ALT and AST levels. No grade 5 TRAEs were observed in this study, while 95.7% (44/46), 47.8% (22/46), and 10.9% (5/46) of patients experienced grades 1-2, 3, and 4 TRAEs, respectively. In present study, the most common grade 3 or higher TRAEs included neutropenia (n=10, 21.7%), thrombocytopenia (n=8, 17.4%), increased AST levels (n=6, 13.0%), and increased ALT levels (n=4, 8.7%). Additionally, immune-related adverse events of any grade were observed in 13 (28.3%) participants, including hepatitis (n=3), dermatitis (n=3) and hypothyroidism (n=7), which could be alleviated and eliminated by treatment interruption or dose modification.

**Table 4:** Treatment Related Adverse Events* (n=46).

	Grade 1-2	Grade 3	Grade 4
Neutropenia	14 (30.4%)	7 (15.2%)	3 (6.5%)
Thrombocytopenia	15 (32.6%)	5 (10.9%)	3 (6.5%)
Nausea	19 (41.3%)	4 (8.7%)	0
Abdominal pain	20 (43.5%)	2 (4.3%)	0
Vomit	17 (37.0%)	3 (6.5%)	0
Fatigue	17 (37.0%)	2 (4.3%)	0
Aspartate aminotransferase increased	12 (26.1%)	6 (13.0%)	0
Alanine aminotransferase increased	10 (21.7%)	4 (8.7%)	0
Hypertension	13 (28.3%)	0	0
Edema	10 (21.7%)	2 (4.3%)	0
Diarrhea	8 (17.4%)	1 (2.2%)	0
Ascites	6 (13.0%)	1 (2.2%)	0
Fever	6 (13.0%)	0	0
Pruritus	6 (13.0%)	0	0
Hand-foot skin reaction	5 (10.9%)	1 (2.2%)	0
Infection	4 (8.7%)	0	1 (2.2%)
Anemia	3 (6.5%)	1 (2.2%)	0
Gastrointestinal bleeding	2 (4.3%)	1 (2.2%)	0
Immune-related adverse event			
Immune-related hypothyroidism	6 (13.0%)	1 (2.2%)	0
Immune-related dermatitis	3 (6.5%)	0	0
Immune-related hepatitis	3 (6.5%)	0	0

*Listed are adverse events, as defined by the National Cancer Institute Common Terminology Criteria (version 4.03), that occurred in at least 5% of patients.

### Overview of the genomic mutation spectrum

**Figure 3:**
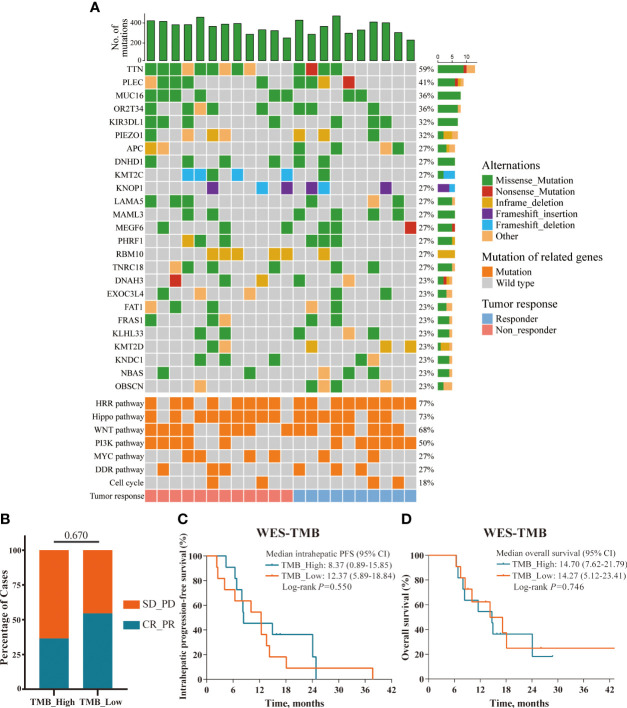
Overview of the genomic mutation spectrum. **(A)** Overview of the genomic mutation spectrum and pathway alterations in the WES cohort (responders, n=10; non-responders, n=12); **(B–D)** TMB status presented no significant correlations with ORR, intrahepatic PFS, and OS; defined by median split.

In the prespecified exploratory analyses, we first evaluated the association between genomic alteration and clinical response to this triple combination therapy in 22 patients with adequate pre-treatment tumor biopsies for WES (10 responders defined as having a complete and partial response, and 12 non-responders defined as having a stable and progressive disease). **Figure 3A** depicted the genetic alterations and frequencies in the entire cohort, including the most commonly altered genes and cancer-related pathways. Unfortunately, none of the genomic mutation or pathway alteration exhibited significant associations with clinical response or survival in univariable analysis (data not shown). We next analyzed the clinical relevance of TMB in the 22 patients with available data. According to the median split, there was no significant correlation between the level of TMB and clinical response, intrahepatic PFS, or OS (**Figures 3B–D**).

### Transcriptome analyses in responders and non-responders

**Figure 4:**
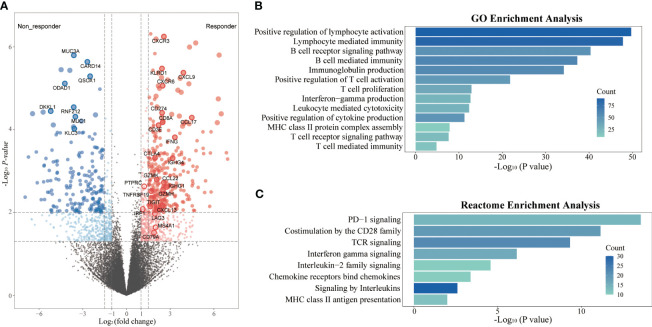
Transcriptome analyses in responders and non-responders. **(A)** Differential expression genes between responders (n=9) and non-responders (n=9) in the RNAseq cohort; **(B)** GO enrichment analysis of differential expression genes; **(C)** REACTOME pathway enrichment analysis of differential expression genes.

**Figure 5:**
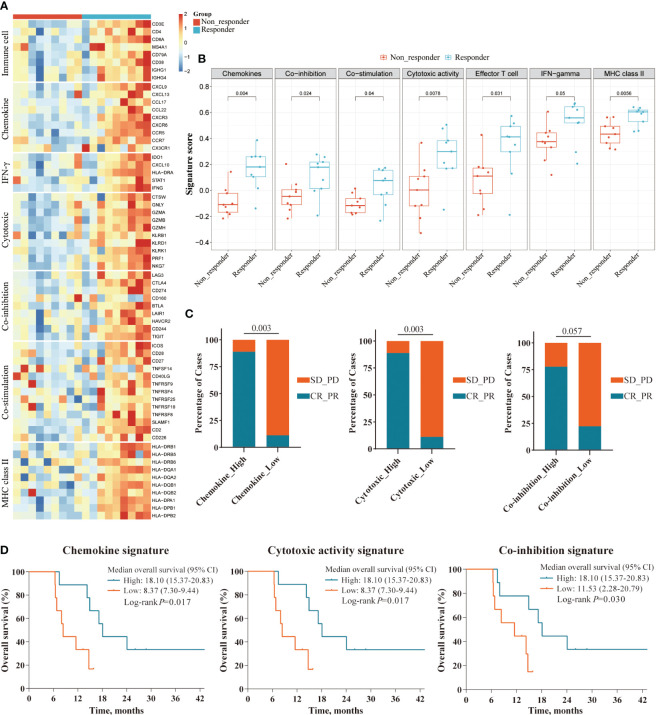
Survival analyses of immune-related gene signatures in responders and non-responders. **(A)** Heatmap showing the expression level of gene signatures representing pre-existing antitumor immunity between responders and non-responders in the RNAseq cohort; **(B)** Boxplot showing the expression scores of gene signatures between responders and non-responders in the RNAseq cohort; **(C, D)** Superior ORR and OS were associated with higher expression level of three gene signatures: Chemokine, Cytotoxic activity, and Co-inhibition signature, defined by the median split.

To further assess the impact of tumor immune microenvironment (TIME) on clinical response to this triple combination therapy, we performed transcriptome analyses in 18 patients (9 responders, 9 non-responders). Genome-wide differentially expressed gene analyses identified multiple genes representing pre-existing antitumor immunity as the top features associated with tumor response, including those immune cell-related genes (*PTPRC*, *CD3E*, *CD8A*, *MS4A1*, and *IGHG4*); cytotoxic T cell signature (*IFNG*, *GZMH*, *GZMK*, *KLRD1*, and *IRF1*); chemokines (*CXCL13*, *CXCL9*, *CCL17*, and *CCL22*), and immune checkpoint target genes (*CD274*, *CTLA4*, *LAG3*, and *TIGIT*, **Figure 4A**). Gene Ontology (GO) enrichment analyses further confirmed that multiple adaptive and innate immunity pathways, including lymphocyte activation, B cell mediated immunity, T cell proliferation, interferon-gamma production and T cell receptor signaling pathway, were significantly enriched in patients that responded to this triple combination (**Figure 4B**). We obtained the similar results in the REACTOME pathway analyses, which were also concentrated in adaptive immunity and chemokine signaling pathways (**Figure 4C**). We next assessed the expression levels of several gene signatures representing pre-existing antitumor immunity and evaluated their association with the clinical outcomes. Notably, we observed that the expression scores of six gene signatures were significantly higher in responders than that in non-responders, namely chemokines (*p* = 0.004), MHC class II signature (*p* = 0.006), cytotoxic activity signature (*p* = 0.008), co-inhibition signature (*p* = 0.024), effector T cell (*p* = 0.031) and co-stimulation signature (*p* = 0.040; **Figures 5A, B**). Furthermore, we observed that iCCA patients with higher expression levels of chemokine, cytotoxic activity, and co-inhibition signatures exhibited improved ORRs (chemokine: 88.9% vs. 11.1%, *p* = 0.003; cytotoxic activity: 88.9% vs. 11.1%, *p* = 0.003; co-inhibition: 77.8% vs. 22.2%, *p* = 0.057) and superior OS (chemokine: 18.10 vs. 8.37 months, *p* = 0.017; cytotoxic activity: 18.10 vs. 8.37 months, *p* = 0.017; co-inhibition: 18.10 vs. 11.53 months, *p*=0.030; **Figures 5C, D**).

**Figure 6:**
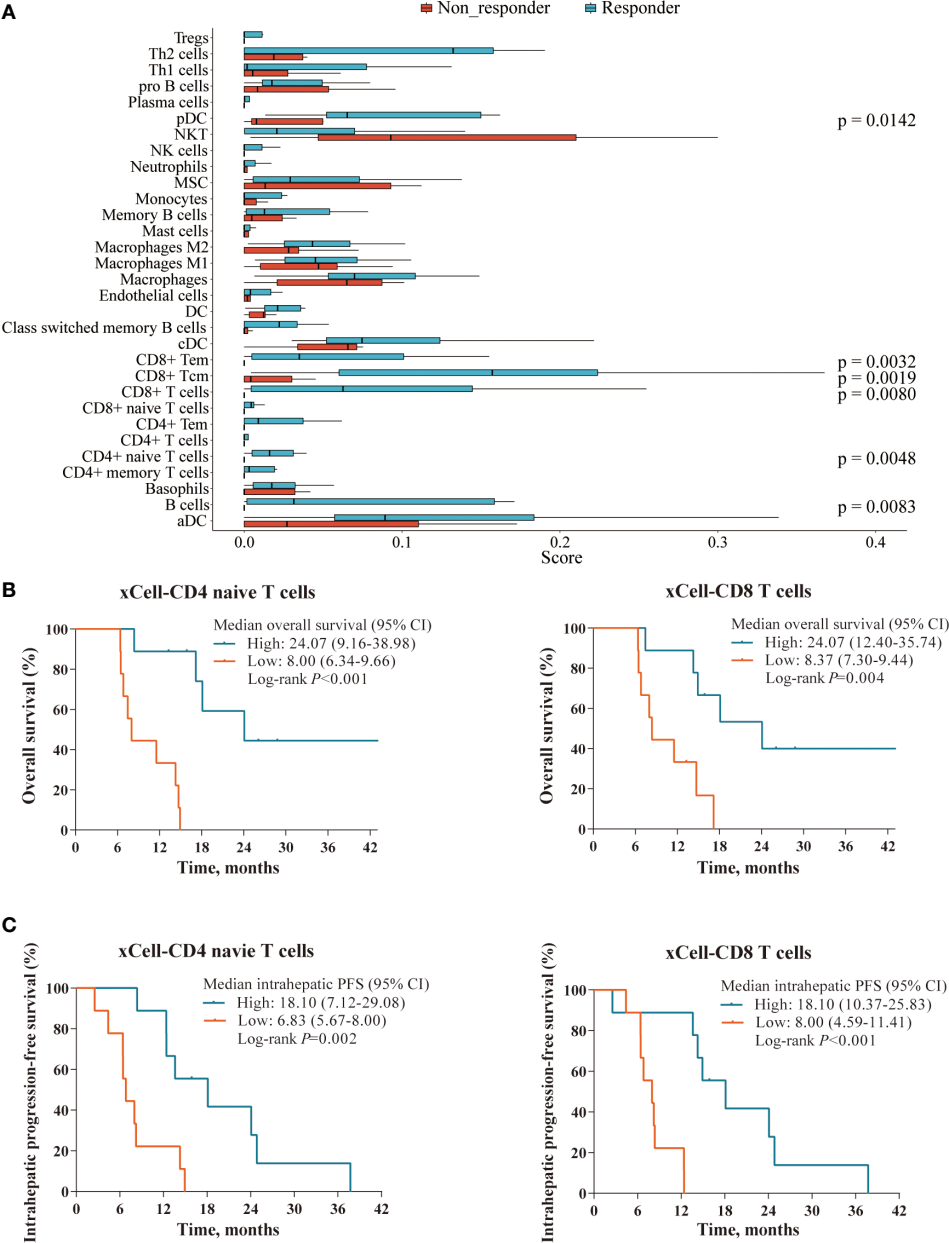
Immune cell profile analyses in the RNAseq cohort. **(A)** Boxplot showing the abundance of several immune cells composition between responders (n=9) and non-responders (n=9) in the RNAseq cohort, deconvolved by xCell algorithm. **(B, C)** Superior OS and intrahepatic PFS were associated with higher levels of tumor-infiltrating lymphocytes defined xCell deconvolution analysis: CD4 naïve T cells (left panel) and CD8 T cells (right panel).

Subsequently, we assessed the abundance of immune cells to further investigate the TIME using xCell deconvolution analysis (25). And we observed that a higher presence of several immune subsets, including CD8^+^ and CD4^+^ T cells, B cells and dendritic cells, also seemed to be associated with better response (**Figure 6A**). Further survival analysis showed that high levels of tumor-infiltrating lymphocytes (TILs) were associated with markedly longer survival outcomes in OS (CD4 naïve T cells: 24.07 vs. 8.00 months, *p* < 0.001; CD8 T cells: 24.07 vs. 8.37 months, *p*=0.004; **Figure 6B**) and intrahepatic PFS (CD4 naïve T cells: 18.10 vs. 6.83 months, *p*=0.002; CD8 T cells: 18.10 vs. 8.00 months, *p* < 0.001; **Figure 6C**).

**Figure 7:**
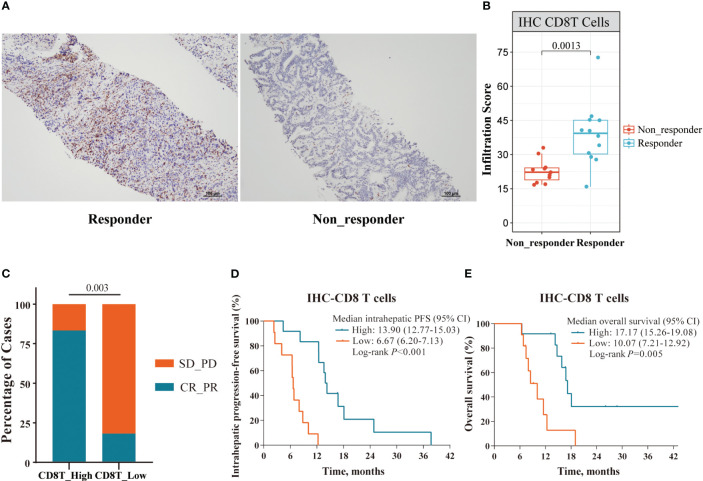
Immune cell profile analyses in the IHC cohort. **(A)** Representative IHC staining images of responder and non-responder; **(B)** The Boxplot showing the significant differences with a density of tumor-infiltrating CD8^+^ T cells between responders (n=12) and non-responders (n=11) in the IHC cohort; **(C–E)** Superior ORR, intrahepatic PFS and OS were associated with higher density of tumor-infiltrating CD8^+^ T cells, defined by median split.

We next analyzed the density of tumor-infiltrating CD8^+^ T cells and their association with clinical outcomes (IHC cohort: 12 responders and 11 non-responders). Consistent with our genomic findings, IHC analysis of 23 tumor samples showed that responders exhibited a higher density of tumor-infiltrating CD8^+^ T cells than non-responders (*p* = 0.0013, **Figures 7A, B**). Patients with a higher density of tumor-infiltrating CD8^+^ T cells exhibited improved ORR (83.3% vs. 18.2%, *p* = 0.003), OS (17.17 vs. 10.07 months, *p* = 0.005) and intrahepatic PFS (13.90 vs. 6.67 months, *p* < 0.001; **Figures 7C–E**).

## Discussion

To the best of our knowledge, this retrospective study is the first to report the efficacy and safety of a novel triple combination therapy with FOLFOX-HAIC, lenvatinib plus PD-1 inhibitor, which yielded a promising antitumor activity with manageable safety profiles in patients with locally advanced and metastatic iCCA.

Many efforts have been made to explore the clinical efficacy of PD-1 inhibitors in advanced BTCs with little success. Previous clinical trials showed the limited antitumor activity of ICIs monotherapy in advanced iCCA, with a median OS of 5.2-7.4 months and ORR of less than 15% (26, 27). However, TOPAZ-1 trial showed that durvalumab plus GemCis provided significant survival benefits compared to GemCis (median OS: 12.8 vs. 11.5 months) (13), which is also recommended as one of preferred first-line treatments for advanced BTCs, including iCCA. In addition, iCCA was characterized by abnormal activation of VEGF and FGFR signaling pathway (28, 29), which are the primary targets of lenvatinib. And the survival benefits were further demonstrated in a recent phase II trial combining toripalimab with lenvatinib and GEMOX, which yielded a high ORR (80%) and a median OS of 22.5 months for advanced iCCA (30). These data had provided definite evidence for the reasoning of chemotherapy, lenvatinib plus PD-1 inhibitor as a combo to treat advanced iCCA.

In our previous publication, we demonstrated lenvatinib, toripalimab and FOLFOX-HAIC exhibited encouraging antitumor activity for advanced HCC (20). These advantages were attainable due to the specific administration patterns of HAIC. FOLFOX-HAIC could deliver chemotherapeutic agents directly into tumor-associated arterial branches, and increase local drug concentrations (31), thus providing stronger antitumor efficacy and lower systemic toxicities than systemic therapies (32). In the current study, we focused on the clinical effects and safety of this similar triple combination for pure advanced iCCA.

Our results displayed potential synergistic effect and promising preliminary efficacy results of this triple combination therapy, with an overall ORR of 47.8%, DCR of 91.3%, median PFS of 9.40 months (95% CI, 5.28-13.52) and median OS of 16.77 months (95% CI, 14.20-19.33). This combination therapy resulted in a higher ORR and better mPFS for advanced iCCA than standard chemotherapy or PD-1 inhibitors plus chemotherapy, thus providing a robust antitumor effect and allowing a proportion of patients to achieve downstaging and conversion to radical resection. Notably, the patient population in this study might be considered to have a poor prognosis because all patients included in this study had stage IIIA disease or higher at baseline, presented with a higher intrahepatic tumor burden. Additionally, we demonstrated that FOLFOX-HAIC played an important role in shrinkage of hepatic lesions. The response rate of intrahepatic lesions was 52.2% per RECIST and 63.0% per mRECIST, including 4 patients achieved complete response based on mRECIST criteria.

TRAEs observed with this combination therapy are consistent with those reported in previous trials (20, 24, 33). The most common TRAEs were neutropenia, thrombocytopenia, abdominal pain, nausea, vomiting, and elevated serum ALT or AST level. Hematologic toxicity was the main grade 3 and higher TRAEs in this study, which were tolerable and manageable. Furthermore, immune-related adverse events of any grade were observed in 13 (28.3%) patients, including hepatitis, dermatitis, and hypothyroidism, which were associated with PD-1 inhibitors as reported in the previous studies (34, 35).

Although this combination significantly improved clinical outcomes, unfortunately, small proportion of patients remained unresponsive. It is therefore important to identify biomarkers to predict which patients could benefit most. By integrating transcriptomic, genetic, and IHC analyses of primary tumors, we characterized the molecular correlates of clinical response and resistance to this triple combination therapy. Surprisingly, gene sequencing result demonstrated no significant correlations of gene mutations, pathway alterations, or TMB with clinical response and survival outcomes. Moreover, there have been contradictory results about the predictive value of TMB status for immunotherapy. Exploratory analysis of prospective trials revealed that higher TMB was associated with superior survival and objective response in advanced solid tumor (36–38). However, several studies evaluating combined-immunotherapy showed that TMB status did not show any predictive value for efficacy in patients with advanced BTC, NSCLC and ESCC (39–42). It is plausible that giving chemotherapy with immunotherapy could have confounded the utility of TMB. Our results showed that neither objective response nor survival differed significantly on the basis of TMB status, highlighting that TMB may not be an effective biomarker for predicting the clinical benefit of this combination therapy.

A central finding from our study is that the presence of pre-existing immunity (higher expression of specific immune-related signatures, and intra-tumoral CD8^+^ T cell density) in baseline tumor tissues was associated with better clinical outcomes with this triple combination. Consistent with the features of ‘immune-hot’ tumor, immune cell-related genes, chemokines, and immune checkpoint targets were found to be upregulated in the responders. Biomarkers of inflammation and inflammatory gene signatures were indeed reported to be associated with response to immunotherapy (39, 43). Our study also observed significant correlations between immune-related signatures (higher expression level of cytotoxic activity, co-inhibition, and co-stimulation signatures) with improved clinical outcomes, suggesting the predominant TIME characteristics of responsive iCCA. It is worth mentioning that, as a crucial prognostic indicator, TILs exhibit great predictive power for survival in solid cancers (44, 45). Moreover, our study further confirmed its value by showing that a higher baseline density of CD8^+^ T cells in the TIME was an indicator of treatment activity, which was associated with significantly superior clinical benefits. Taken together, pre-existing antitumor immunity in baseline tumor might be predictive biomarkers for the triple combination therapy. However, confirmation in a larger study population is required to verify our preliminary results.

This study had several limitations. First, this was a retrospective and nonrandomized study with a limited sample size, for which the retrospective design and nonrandomized nature made it vulnerable to a variety of potential biases. These findings in the study needed prospective randomized controlled trials to verify. Second, the follow-up time was relatively short for OS, but was sufficient for short-term efficacy (PFS and tumor responses). Therefore, we chose PFS as the primary endpoint in this study, which could eliminate the confounding effect of subsequent therapy and reflected the efficacy more accurately. Finally, due to the relatively small number of tissue specimens, exploratory research in this study did not have strong statistical power to draw a definite conclusion on the predictive role of these candidate biomarkers.

## Conclusion

In conclusion, this retrospective study provided preliminary evidence that FOLFOX-HAIC in combination with lenvatinib and PD-1 inhibitor showed a promising antitumor activity with manageable safety profiles in advanced iCCA. Moreover, our results also revealed new perspectives on potential biomarkers for clinical efficacy. These findings warrant further validation in a large randomized clinical trial.

## Data availability statement

The raw data generated in this study have been deposited in the Gene Expression Omnibus (GEO) database, with the accession number as GSE255058.

## Ethics statement

The studies involving humans were approved by institutional review board of Sun Yat-sen University Cancer Center. The studies were conducted in accordance with the local legislation and institutional requirements. The participants provided their written informed consent to participate in this study.

## Author contributions

YH: Conceptualization, Data curation, Formal Analysis, Investigation, Visualization, Writing – original draft. ZD: Data curation, Formal Analysis, Methodology, Software, Visualization, Writing – original draft. AK: Data curation, Funding acquisition, Investigation, Methodology, Writing – original draft. MH: Conceptualization, Data curation, Funding acquisition, Investigation, Writing – original draft. HL: Data curation, Formal Analysis, Methodology, Writing – original draft. ZL: Formal Analysis, Investigation, Methodology, Writing – original draft. DW: Data curation, Investigation, Methodology, Writing – original draft. LH: Data curation, Methodology, Writing – original draft. QL: Data curation, Investigation, Methodology, Writing – original draft. LX: Conceptualization, Investigation, Supervision, Validation, Writing – original draft. MS: Conceptualization, Data curation, Funding acquisition, Methodology, Project administration, Supervision, Writing – review & editing.

## Glossary

**Table:**

iCCA	Intrahepatic Cholangiocarcinoma
HCC	Hepatocellular Carcinoma
GemCis	Gemcitabine plus cisplatin
GEMOX	Gemcitabine and oxaliplatin
HAIC	Hepatic Arterial Infusion Chemotherapy
BTCs	Biliary Tract Cancers
TACE	Transarterial Chemoembolization
FOLFOX	Oxaliplatin, leucovorin plus 5-fluorouracil
ICIs	Immune Checkpoint Inhibitors
TKIs	Tyrosine Kinase Inhibitors
PD-1	Programmed Cell Death Protein-1
CT	Computed Tomography
MRI	Magnetic Resonance Imaging
RECIST	Response Evaluation Criteria in Solid Tumors
OS	Overall Survival
PFS	Progression-Free Survival
ORR	Objective Response Rate
DCR	Disease Control Rate
WES	Whole Exome Sequencing
IHC	Immunohistochemistry
HR	Hazard Ratio
CI	Confidence Interval
IQR	Interquartile Range
SD	Standard Deviation
TRAEs	Treatment-related Adverse Events
TMB	Tumor Mutation Burden
HRR	Homologous Recombination Repair
DDR	DNA Damage Response
TILs	Tumor-infiltrating Lymphocytes
TIME	Tumor Immune Microenvironment.
